# Mucoadhesive Vaginal Discs based on Cyclodextrin and Surfactants for the Controlled Release of Antiretroviral Drugs to Prevent the Sexual Transmission of HIV

**DOI:** 10.3390/pharmaceutics12040321

**Published:** 2020-04-02

**Authors:** Fernando Notario-Pérez, Araceli Martín-Illana, Raúl Cazorla-Luna, Roberto Ruiz-Caro, Aitana Tamayo, Juan Rubio, Veiga María-Dolores

**Affiliations:** 1 Departamento de Farmacia Galénica y Tecnología Alimentaria, Facultad de Farmacia, Universidad Complutense de Madrid, Plaza Ramón y Cajal s/n, 28040-Madrid, Spain; fnotar01@ucm.es (F.N.-P.); aracelimartin@ucm.es (A.M.-I.); racazorl@ucm.es (R.C.-L.); rruizcar@ucm.es (R.R.-C.); 2 Instituto de Cerámica y Vidrio, Consejo Superior de Investigaciones Científicas, Calle Kelsen 5, 28049-Madrid, Spain; aitanath@icv.csic.es (A.T.); jrubio@icv.csic.es (J.R.)

**Keywords:** coitally-dependent microbicide, dapivirine, freeze-dried gels, human immunodeficiency virus, 2-hydroxypropyl-β-cyclodextrin, mucoadhesive vaginal discs, surfactant, tenofovir

## Abstract

The strategies for developing vaginal microbicides to protect women against human immunodeficiency virus (HIV) sexual transmission are constantly changing. Although the initial dosage forms required daily administration to offer effective protection, the trend then moved towards sustained-release dosage forms that require less frequency of administration in order to improve women’s compliance with the treatment. Nevertheless, another possible strategy is to design on-demand products that can be used in a coitally-dependent manner and only need to be administered immediately before intercourse to offer protection. Vaginal discs based on freeze-dried hydroxypropylmethyl cellulose gels have been developed for this purpose, containing two surfactants, i.e., sodium dodecyl sulphate and polysorbate 60, alone or in combination with 2-hydroxypropyl-β-cyclodextrin, to achieve a formulation capable of incorporating both hydrophilic and lipophilic drugs. Several studies have been carried out to evaluate how the inclusion of these substances modifies the structure of gels (viscosity and consistency studies) and the porosimetry of the freeze-dried discs (scanning electron microscopy micrographs, mechanical properties, swelling behaviour). The drug release and mucoadhesive properties of the discs have also been evaluated with a view to their clinical application. The systems combining sodium dodecyl sulphate and 2-hydroxypropyl-β-cyclodextrin were found to be adequate for the vaginal administration of both Tenofovir and Dapivirine and also offer excellent mucoadhesion to vaginal tissue; these discs could therefore be an interesting option for a coitally-dependent administration to protect women against HIV transmission.

## 1. Introduction

Human immunodeficiency virus (HIV) infection continues to be a major health problem worldwide, although in recent years great strides have been made in access to antiretroviral therapy and in slowing the incidence of new infections [1]. According to the latest data from the World Health Organization, in 2018 there were 37.9 million people living with HIV, of which 1.7 million were infected in that year. Women deserve a special mention, as it is estimated that every day about 860 young women (between 15–24 years old) become infected. These data are especially worrying in sub-Saharan Africa, where 80% of young people suffering from the infection are women [2].

Consequently, research into vaginal microbicides has soared in recent decades. These microbicides are formulations with a topical application that can be used by women without the need for men’s cooperation. They need to be effective in preventing the transmission of the virus, safe (with a minimal effect on the integrity of the cervicovaginal epithelium), and comfortable for women [3,4]. Although numerous strategies were initially evaluated to develop vaginal microbicides (surfactants, acidifiers, polyanions, monoclonal antibodies, etc.), it has now been observed that only microbicides containing antiretroviral drugs can actually inhibit in vivo infection [4,5,6].

Undoubtedly, the two most widely studied antiretroviral drugs for the manufacture of vaginal microbicides are Tenofovir (TFV) and Dapivirine (DPV), given the good results obtained in clinical trials [5]. TFV is a nucleoside reverse transcriptase inhibitor (NRTI) that has been included as a microbicide in numerous pharmaceutical forms (gels, films, rings, tablets) [7,8]. It is notable that the first microbicide to demonstrate protective efficacy in clinical trials was a 1% TFV gel (CAPRISA 004 trial), which was shown to reduce HIV infections by 39% [9]. In contrast, DPV is a non-nucleoside reverse transcriptase inhibitor (NNRTI); it is a non-competitive inhibitor that binds to reverse transcriptase at an allosteric site and causes a conformational change in the enzyme [10]. Although films, gels, and tablets loaded with this drug have also been developed, vaginal rings are particularly worth highlighting, since two clinical trials conducted with this formulation (The Ring Study and ASPIRE trial) demonstrated protective efficacy against HIV [11,12]. Although the two drugs have proven potential, it should be noted that while TFV has demonstrated its efficacy in vaginal gels (as a hydrophilic drug, strategies have been sought to accelerate its dissolution after administration), vaginal rings have generally been chosen for long-term release in the case of DPV (made possible thanks to the lipophilicity of the drug, which is not very soluble in vaginal fluid). It is difficult to find a suitable formulation in which the two drugs could be incorporated, although some researchers have succeeded in developing both vaginal films and rings that combine the two active principles [13,14].

Among the different dosage forms developed for the vaginal administration of microbicides (gels, creams, tablets, films, and vaginal rings [3]), gels are undoubtedly the most frequently used, since they allow a rapid release of the active substance (which enables their use on demand prior to sexual intercourse), and they are also economical and easily for women to apply [5]. However, the main problems cited by users are leakage and messiness after administration [15]. Freeze-drying is one strategy to solve this problem with semi-solid formulations, involving a process in which water is removed from the pharmaceutical form [16,17]. These freeze-dried gels are equally convenient to administer and allow the rapid release of the active substance, since they can quickly rehydrate in the presence of vaginal fluid [18]. This would also make it possible to minimize and even eliminate the inconvenience caused by the leakage of the gels.

Gels for vaginal administration can be prepared in a wide variety of natural, synthetic or semi-synthetic polymers [19], among which it is worth highlighting cellulose derivatives for their extreme versatility. Since it is a semi-synthetic polymer, the cellulose can be modified by substituting different functional groups in order to obtain a wide variety of polymers (hydroxypropylmethyl cellulose (HPMC), methylcellulose, hydroxypropyl cellulose, hydroxyethyl cellulose, ethyl cellulose, etc.), all of which can be used to prepare vaginal gels, depending on the desired characteristics in the formulation [20,21,22].

When formulating gels with poorly water-soluble drugs such as DPV, excipients such as surfactants or cyclodextrins must be added to ensure the solubility of the drug in the aqueous medium. Cyclodextrins have been shown not only to increase drug solubility but also to have the potential to modulate the release rate of the active substance, thus offering a strategy for the development of sustained-release formulations [23]. It has also been observed that the structure of gels based on cellulose derivatives can be modified by the incorporation of cyclodextrins, which vary the arrangement of the polymer network [24]. Finally, there are references to possible interactions when surfactants are combined with cyclodextrins [25].

Vaginal discs have been developed in this work. These discs consist of freeze-dried gels manufactured with a flat and circular shape. Thus, they provide all the previously mentioned advantages of freeze- dried systems for vaginal administration. Moreover, it must be mentioned that the shape of the formulation has been designed with a view of vaginal films, trying also to provide the advantages of this dosage form. The flat shape will provide a more comfortable administration, while the large surface would allow better mucoadhesion and faster water capture.

Based on this background, the aim of this work is to prepare vaginal discs obtained by freeze-drying gels based on HPMC. These gels incorporate a water-soluble antiretroviral drug (TFV) or a poorly water-soluble drug (DPV). Surfactants (both neutral and anionic in nature) and cyclodextrins will also be included to determine their possible influence on the gel structure, to determine how their presence affects freeze-dried gels, and particularly to assess their ability to modify the drug release. The objective is to develop a formulation that is comfortable for women to administer and that ensures the rapid dissolution of the drugs, therefore allowing their discreet use on demand immediately before sexual intercourse. Thus, the developed microbicide could improve the main problem observed in clinical trials with these drugs, which was the lack of adherence to treatment by women.

## 2. Materials and Methods

### 2.1. Materials

Tenofovir (TFV, lot: FT104801501) was provided by Carbosynth Limited (Berkshire, UK). Dapivirine (DPV; lot: 60416PIL04) was kindly provided by the International Partnership for Microbicides (IPM, Silver Spring, MD, USA). Hydroxypropylmethyl cellulose—Methocel® K4M (HPMC; lot: SK27012N11) was kindly supplied by Colorcon Ltd. (Kent, UK). 2-Hydroxypropyl-β-cyclodextrin (2HPβCD; lot: BCBQ9423V), Polysorbate—Tween® 60 (P60; lot: MKBT3178V), and sodium dodecyl sulphate (SDS; lot: STBJ1530) were acquired from Sigma–Aldrich (St. Louis, MO, USA). Technical-grade water (lot: 9V011540) was acquired from Panreac (Barcelona, Spain).

All other reagents in this study were of analytical grade and were used without further purification. Demineralised water was used in all cases, except in the preparation of the gels, when technical-grade water was used.

### 2.2. Gel Preparation

Six batches of unloaded gels were manufactured. The reference batch was a 2% HPMC gel, prepared to evaluate the structure of the gel when no other component was added. Three HPMC-based gels including 10% of 2HPβCD, SDS (anionic surfactant) or P60 (neutral surfactant) were then manufactured in order to determine how the addition of these components modified the polymer network and gel properties. To evaluate possible interactions between surfactants and cyclodextrins, two extra HPMC gels were also prepared containing 10% of 2HPβCD and 10% of SDS or P60. Finally, the same six batches containing 0.25% TFV or 0.125% DPV were prepared as drug-loaded gels. The composition of the gels was proportional to the dose per disc desired, which is presented in Table 1.

Gels were prepared by dissolving the cyclodextrin and/or surfactant in double-distilled water, then adding the drug until its dissolution (or until its homogeneous dispersion in batches with DPV but without surfactant, when the active principle was not dissolved). Finally, HPMC was added, and the system was stirred until its complete gelation. The gels were left to rest for 72 h prior to their characterisation to guarantee their complete homogenization. A bulk gel of each batch was prepared and characterised. Subsequently, each vaginal disc of the same batch was prepared from this gel.

### 2.3. Gel Characterisation

#### 2.3.1. Viscosity

The viscosity of the gels was determined using a viscosimeter (Visco Elite, Fungilab S.A., Sant Feliu Llobregat, Spain): 100 mL of the gel at room temperature was placed in a 4 cm diameter tube. The viscosity (mPa·s) was measured every 10 s for 180 s, using the appropriate probe (L2, L3 or L4). However, the values were observed to be constant after the first 20 s, so it was decided to use values obtained after 30 s of the test. This study was carried out on the bulk gel prepared for each batch.

#### 2.3.2. Penetration and Detachment Work

A study was carried out using a Texture Analyser (TA.XT*plus*, Stable Micro Systems, Surrey, UK) to determine the penetration work (a measure of gel consistency) and the detachment work (related to gel adhesiveness). The characterisation was done using the protocol of Martín-Illana et al., with a 2 cm diameter probe and a 5 kg load cell [17]. Specifically, 80 mL of the evaluated gel was placed in a 100 mL beaker. The probe was located 60 mm above the base of the equipment (10 mm above the gel surface) and was lowered onto the gel at a speed of 0.5 mm/s after an activation force of 2 g. In the test, the probe penetrated the gel to a distance of 1.5 cm and was then returned to its initial position. The force required by the probe to penetrate and detach the gel was recorded. The test was performed nine times for each batch. The results were compared with a paired *t*-test, with α = 0.05.

### 2.4. Vaginal Disc Preparation

**Figure 1:**
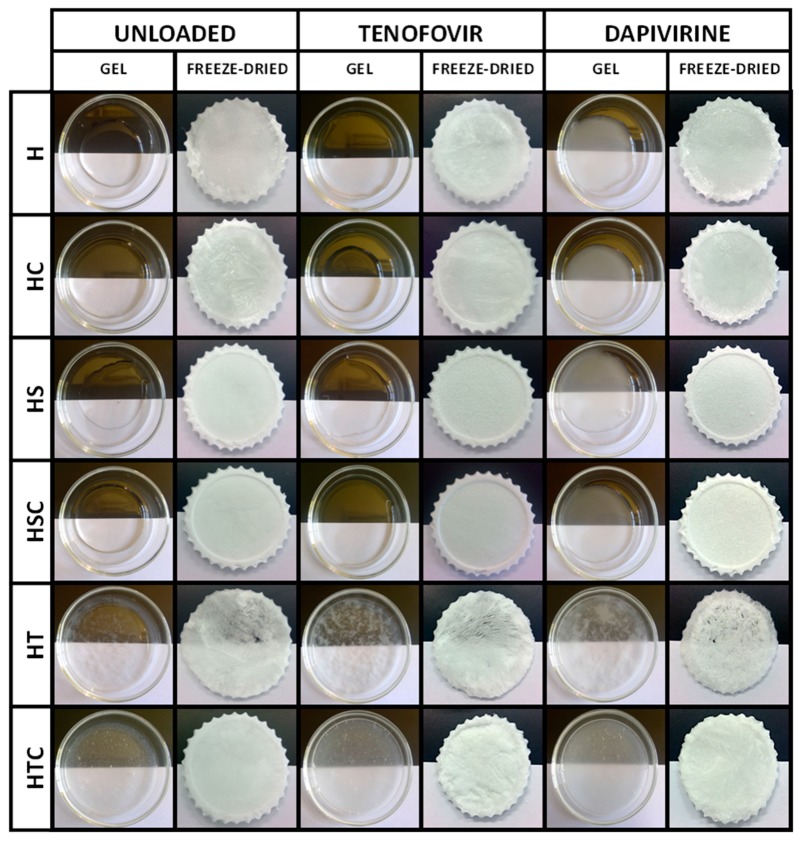
Appearance of each batch of fresh and freeze-dried gels.

Vaginal discs were prepared by freeze-drying the previously prepared gels (Figure 1). The gels were dosed on silicone templates with a diameter of 5 cm in a sufficient amount to obtain a final dose of 10 mg TFV or 5 mg DPV for each disc. The amount of TFV was equivalent to the dose in gels and fast-release films already tested in clinical trials [9]. The amount of DPV included was higher than the dose required to achieve inhibitory concentrations, in order to characterise the amount of this drug that could be dissolved with the applied solubilization technologies.

Unloaded vaginal discs were also prepared by adding the required amount of gel to obtain discs with the same composition as their equivalent drug-loaded disc (except for the active principle, obviously). A Lio-Labor® freeze-dryer (Telstar, Barcelona, Spain) was used to obtain the discs, using a freezing temperature of –45 °C, a sublimation pressure of 4.5 × 10^−4^ atm and a sublimation temperature between –45 °C and 25 °C [17,26]. The composition of all the prepared batches is shown in Table 1. The visual appearance of the gels and discs can be seen in Figure 1.

### 2.5. Vaginal Disc Characterisation

#### 2.5.1. Apparent Density Calculation

To evaluate the uniformity of each batch of vaginal discs, the discs were weighed on a precision balance and the height was measured with the Texture Analyser. This was done by placing the 2 cm-diameter probe above the vaginal disc at a distance of 5 cm and lowering it at a speed of 0.1 mm/s until a force of 10 g was detected, at which point it stopped. The height of the discs was determined as the difference between the distance of the probe at the start of the trial (5 cm) and the distance at which it stopped. Apparent density of the vaginal discs was determined from the disc diameter (5 cm) and the height values. These tests were done with eight samples of drug-loaded discs and five samples of unloaded discs. Since the discs have a cylindrical flat shape, Equation (1) was used to calculate their apparent density, where *ρ* is the apparent density and *m*, *r,* and *h* represent the weight, the radius (25 mm), and the height of the disc, respectively.

(1) $$\rho =\frac{m}{\pi \cdot r^{2}\cdot h}$$

#### 2.5.2. Porosity Measurement

A vaginal disc from each batch was also characterised by mercury porosimetry using an Autopore II 9215 (Micromeritics Corp., Norcross, GA, USA). Pose size distribution (PSD) was determined, and the corresponding mean pore size (Dp), pore volume (Vp), apparent density (ρ), and porosity (P) were calculated based on this, assuming the pores have a cylindrical shape [27]. Cumulative and incremental pore size distribution graphs were plotted with these data.

#### 2.5.3. Scanning Electron Microscopy (SEM) Micrographs

Micrographs of the surface of the vaginal discs were captured using a scanning electron microscope (JEOL JSM-7600F, JEOL Ltd., Tokyo, Japan) at an accelerating voltage of 5 kV. One disc from each batch was fixed on the microscope sample holder and coated for 90 s in a high-vacuum atmosphere with a gold sputter module. Micrographs were captured at ×25, ×50 and ×100 magnifications.

#### 2.5.4. Mechanical Properties: Deformation and Resistance to Fracture

These tests were done using the Texture Analyser. For the deformation test, the equipment was installed with a 5 kg load cell, and a 2 cm cylindrical probe was placed at an initial height of 5 cm. The vaginal disc was placed on the base of the Texture Analyser, and the probe was lowered onto the disc at a rate of 0.5 mm/s. When an activation force of 2 g was detected, the Texture Analyser began recording the force applied until the vaginal disc was deformed by 1 mm. The probe then returned to its initial position before descending again in a cyclical mode, until ten deformation cycles had been completed [18]. The test was performed with four samples of each batch. The deformation force was established as the force required to deform the disc by 1 mm in the first cycle, and the loss of deformability was calculated as the difference between the force applied in the first and the tenth cycle, as a percentage.

Elasticity and resistance to fracture of the discs were determined with a method previously used to evaluate vaginal films [28]. The puncture test was conducted with a probe with a circular head 5 mm in diameter. The film was fixed on a rigid support with a hole, and the probe was lowered through this hole at a speed of 0.5 mm/s. The force applied and the distance the disc was deformed were recorded, and the disc burst strength was established as the force at which the probe caused the disc to rupture. This test was also done in quadruplicate.

#### 2.5.5. Swelling Behaviour

Once administered, the vaginal disc captures vaginal fluid to reconstitute the gel. To evaluate how this process takes place, swelling studies were done using simulated vaginal fluid (SVF), prepared as described by Owen and Katz [29]. The vaginal discs were fixed to stainless steel discs using an acrylic adhesive and placed at the bottom of a 250 mL beaker, immersed in SVF. The beakers were inserted in an oscillating water bath (Selecta® Unitronic320 OR, Barcelona, Spain) at 37 °C and 15 opm to simulate conditions in the vaginal environment. At predetermined times—0.5, 1, 2, 3, 4, 5, 6, 24 h, and each 24 h from that point—the discs were removed from the medium and weighed to measure the water capture and erosion in the formulation. Each batch was tested in triplicate. The swelling ratio (SR) was calculated as the percentage of weight at each time compared to the initial weight of the dried disc.

#### 2.5.6. Drug Release Studies

In vitro drug release was tested to evaluate the drug’s dissolution profile in the vaginal environment. Each vaginal disc was placed at the bottom of a borosilicate glass flask with 80 mL of SVF, and the flasks were inserted in a shaking water bath at 37 °C and 15 opm. At predetermined times—0.5, 1, 2, 3, 4, 5, 6, 24 h, and each 24 h from that point—a 5 mL aliquot was removed and filtered, and the medium was replaced with the same amount of SVF in order to maintain a constant volume. Drug concentration was quantified by UV–visible spectroscopy at wavelengths of 260 nm for TFV and 287.5 nm for DPV, using an Evolution 60S spectrophotometer (Thermo scientific, Waltham, MA, USA). Each batch was tested in triplicate. Batches containing DPV were also evaluated using modified SVF containing 5% SDS [10]. This test evaluates the pattern of DPV release from the vaginal discs when solubility is not limited and compares it with the pattern in SVF.

To evaluate if there were statistical differences among batches, the experimental data were compared by the similarity factor (f_2_). This is a model-independent index that can be calculated by means of Equation (2); *n* is the number of samples, *R_j_* the drug release percentage for the reference batch, *T_j_* the drug release percentage for the test, and *W_j_* a weight factor.

(2) $$f_{2}=50\;\times \;log\left\{{\left[1+\left(\frac{1}{n}\right)\sum_{j=1}^{n}W_{j}\left|R_{j}-T_{j}\right|\right]}^{-0.5}\;\times \;100\right\}$$

#### 2.5.7. Mucoadhesion Time and Force

The adhesion of the vaginal discs to the vaginal mucosa was evaluated with two different methodologies. Mucoadhesion time was determined with a method previously described for the evaluation of vaginal tablets [7]. A portion of freshly excised veal vaginal mucosa obtained from a local slaughterhouse was fixed to a stainless-steel plate with cyanoacrylate adhesive. The vaginal disc was placed over the mucosa, and a force of 500 g was applied for 30 s. The system was placed in a 250 mL beaker, immersed in SVF, and inserted in a shaking water bath (37 °C, 15 opm). The mucoadhesion time was visually observed and established as the time at which the disc detached from the mucosa or completely dissolved in the SFV. This test was performed in duplicate for each batch.

Mucoadhesion force was determined using a Texture Analyser equipped with a 5 kg load cell and a cylindrical probe with a diameter of 1 cm. A sample of vaginal mucosa was fixed on a Petri dish with cyanoacrylate adhesive, and the Petri dish was fixed to the base of the Texture Analyser with double-sided tape. A portion of the vaginal disc was fixed to the probe with double-sided tape and was placed at a height of 40 cm above the mucosa. The probe descended at a speed of 1 mm/s and pressed the mucosa with a force of 500 g for 30 s (the same conditions established in the mucoadhesion time test). After this time, the probe was raised at a speed of 0.1 mm/s, and the force required to detach the disc from the mucosa was recorded [17]. This test was performed in triplicate for each batch. The mucoadhesion force was established as the maximum force required to detach the disc, and the work of mucoadhesion was the area obtained when representing the force required to detach the discs against the time. The results were compared by means of a paired t-test, with α = 0.05.

## 3. Results and Discussion

### 3.1. Gel Characterisation

#### 3.1.1. Viscosity

**Figure 2:**
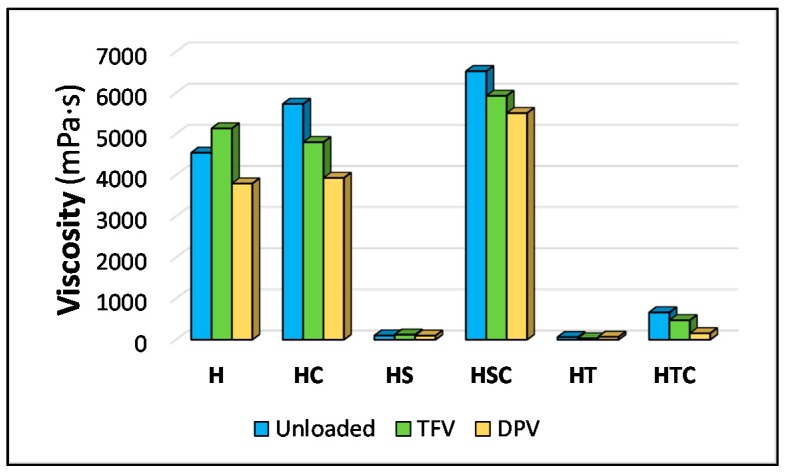
Viscosity of each batch of gels.

Gel viscosity is a basic character that indicates how the structure is affected by the addition of different components. As can be seen in Figure 2, the gel prepared as a reference (H batch) yields a viscosity value close to 4000 mPa·s, which is consistent with the manufacturer’s data since we used Methocel K4M (the viscosity value of a 2% gel at 25 °C should be 4000 mPa·s) [30]. This value is similar to that obtained by other authors when measuring HPMC gels [31]. The high viscosity of this gel suggests it is a good potential candidate to ensure the sustained release of drugs from the freeze-dried gel, as it is able to hydrate quickly and subsequently block any additional uptake of liquids (such as vaginal fluid, which will be required to dissolve the drug so it can be released once the formulation is applied) [32].

It can clearly be seen how the viscosity drops sharply to below 100 mPa·s when a surfactant is added. This effect, which is attributed to the decrease in tension between polymer chains, has been observed for these same surfactants and for many others [33,34,35]. In these batches, the micelles in the surfactants act as a spatial impediment and prevent the free organization of the HPMC polymer chains and their subsequent gelation. In addition, the surfactants are able to reduce the surface tension of water, which is the main component of the gels, and this parameter has a great influence on the viscosity of the gel. The incorporation of 2HPβCD into the HPMC gel increases the viscosity, as expected due to the viscous nature of this component [36,37,38]. However, more striking results are seen when cyclodextrins are combined with surfactants; in the combination of 2HPβCD with polysorbate (HTC batch), a rather low viscosity value is obtained, although higher than for the HT batch, indicating that the character of the surfactant, which reduces the viscosity of the gel, predominates over the presence of cyclodextrins. In this batch, the polysorbate cannot interact with the 2HPβCD molecules because its molecules are too large to penetrate the cyclodextrin cavity, so in the absence of a polysorbate/cyclodextrin interaction, the P60 micelles are maintained and prevent the organisation of the HPMC chains, as observed in the HT batch. In contrast, higher viscosity values are obtained in the combination with SDS (HSC batch) than with either of the two components separately, which is noteworthy since SDS, as a surfactant, greatly reduces viscosity. This value for the measure of viscosity points to a possible interaction of SDS with 2HPβCD, in agreement with previous results in the literature [39,40], and indicating that the surfactant may have entered the cyclodextrin cavity. This would reduce the production of SLS micelles, prevent their exposure to interact with the polymer matrix, and eliminate their effect on the gel viscosity. The organisation of HPMC chains is therefore not modified by the presence of the surfactant, which allows its gelation. The incorporation of the drugs also seems to have an influence on the viscosity of the gels. Thus, TFV-loaded gels have a slightly lower viscosity than unloaded gels—except for reference batches. DPV-loaded gels have an even lower viscosity. This may be related to the interposition of the drug among the HPMC chains, which results in difficult interaction among these chains and prevents the polymer from forming the gel structure as occurred in the unloaded batches.

#### 3.1.2. Penetration and Detachment Work

The mechanical properties of the gels were also characterised through an experiment performed on the Texture Analyser, which can be very valuable for observing how the texture profile of the gels changes according to their composition [41]. The penetration work (which is a measure of the consistency or strength of the gel) and the detachment work (which is a measure of the adhesiveness of the gel) were quantified. 

**Figure 3:**
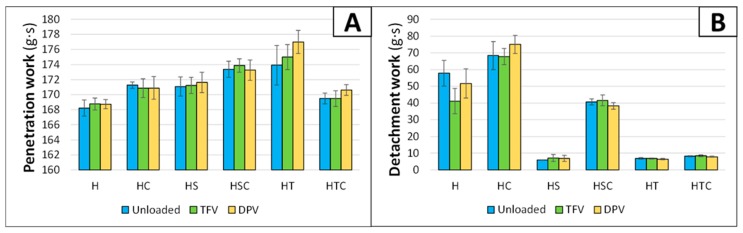
Penetration work (**A**) and detachment work (**B**) for each batch of gels. Mean ± SD are presented (*n* = 9).

The results observed in the penetration work clearly show that there are no significant differences when the drugs are included in the gels (Figure 3A). This was statistically demonstrated with a paired t-test (Table 2). Differences in the consistency of the gels are minimal (all batches have strength values between 168–178 g·s), but still apparent. Reference gel (H batch) has the lowest consistency, and when cyclodextrin or any surfactant is included, the consistency increases. In addition, when SDS and 2HPβCD are combined, the consistency is even higher than when they are included separately. However, the most unexpected result is observed in the combination of polysorbate and 2HPβCD, because although the surfactant alone (HT batch) has the highest consistency, the combination (batch HTC) decreases the gel strength. The visual aspect of this batch, where a phase separation is observed in the gel when it is left at rest, suggests that there is an incompatibility among the components at this concentration, and subsequently the gel loses its structure.

The measurements for the detachment work are clearly related to the viscosity of the gels, since the graph (Figure 3B) is similar to the graph for the viscosity values (Figure 2). Again, there are no significant differences between unloaded and drug-loaded batches (Table 2). While the presence of 2HPβCD slightly increases the adhesiveness of the batches, the addition of a surfactant reduces the detachment work. The presence of the surfactant, as commented previously in the viscosity results, decreases the surface tension of the system. The lower surface tension makes it easier for the probe to detach from the gel, and that is the main reason why the inclusion of a surfactant causes a decrease in the detachment work.

In the cyclodextrin and surfactant combinations, as was observed in the viscosity measurements, it is notable that the inclusion of SDS in batches also containing 2HPβCD (HSC batch) maintains the original adhesiveness of the gel (undoubtedly due to the aforementioned interaction). This can be corroborated with the results of the t-test, as it is observed that there is no difference among batches. The combination of polysorbate and 2HPβCD (HTC batch) shows similar detachment work values to the single inclusion of a surfactant, which was also expected due to the phase separation and loss of structure observed in the gel, although minimal differences are apparent in the statistical analysis that makes us think about a minimal increase of adhesiveness due to the presence of the cyclodextrin.

The effect of incorporating cyclodextrins on the texture profile of the gels was expected, since other authors have also described the increase in the hardness, cohesiveness, strength, and adhesiveness of hydrogels [42]. The decline in adhesiveness with the addition of surfactants has also been evaluated and proven with different methods and is attributed to the lower interaction of the polymer with the probe due to the presence of the surfactant [43]. This also explains why the adhesiveness does not vary when SDS and 2HPβCD are combined (HSC batch); the inclusion of SDS in the cyclodextrin renders it unable to prevent contact between HPMC and the probe and maintains the adhesive properties of the polymer.

### 3.2. Vaginal Disc Characterisation

#### 3.2.1. Apparent Density Calculation

Once the vaginal discs have been manufactured, their appearance and size are analysed to confirm that the manufacturing process is adequate to obtain reproducible discs and to evaluate whether any of the combinations of components undergo alteration during the process. The dimensions of the discs were 50 mm in diameter (determined by the shape of the template used for freeze-drying the gel) and 4.24 ± 0.77 mm in height.

**Figure 4:**
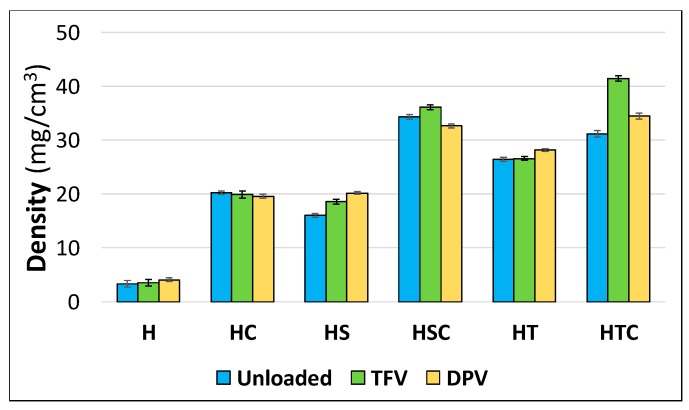
Density values calculated for each batch of discs. Mean ± SD are presented (*n* = 5 for unloaded batches, *n* = 8 for drug-loaded batches).

When determining the apparent density of the discs based on their height and weight, it can be seen that the reference batches (H) have the lowest density. The original gels have similar volumes, which will determine the final volume of the disc, although the substance load in each batch varies considerably. In the reference batches, this load is minimal, only 80 mg of HPMC per disc, alone or in combination with 10 mg TFV or 5 mg DPV, so it was expected that these batches had the lowest density values (Figure 4). In contrast, the density is higher in the batches combining cyclodextrins and surfactants due to the greater load of substances in the discs—400 mg of 2HPβCD and/or surfactant. Among batches containing only cyclodextrin or a surfactant, the batches with polysorbate have a much higher density due to the more compact structure obtained in these discs. Finally, in the batches combining 2HPβCD and a surfactant, the discs containing TFV have a higher density as the drug is internalised more effectively in the gel structure. However, when only surfactants are incorporated in the discs, the DPV-loaded discs have a higher density. This is a great advantage for sustained drug release, since the higher density of freeze-dried systems generally implies a smaller average pore size in the structure [44]. 

#### 3.2.2. Porosity Measurement

**Figure 5:**
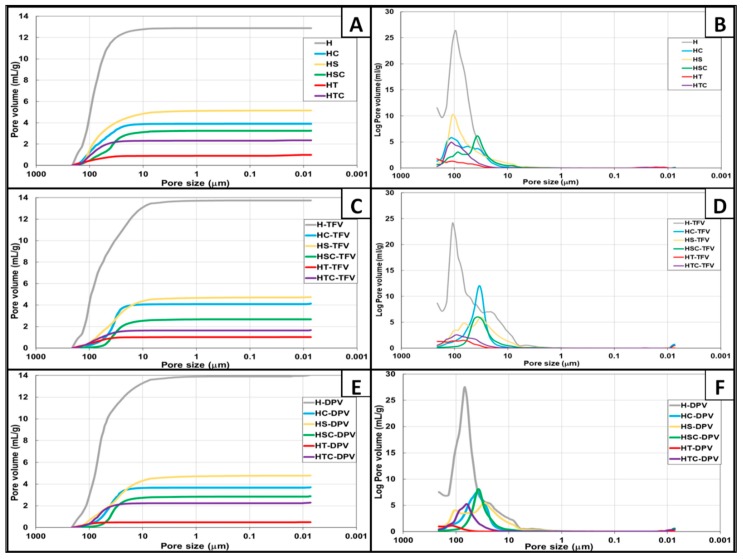
Pore size distributions of vaginal discs, presented as the cumulative volume of unloaded (**A**), TFV (**C**), and DPV (**E**) batches and the logarithm of the differential pore volume of unloaded (**B**), TFV (**D**), and DPV (**F**) batches.

The size and volume of the pores were subsequently analysed through porosity studies. Figure 5A,C,E show that the total pore volume is not significantly modified regardless of whether the batches are unloaded, TFV-loaded or DPV-loaded. However, there are major differences depending on the components in the discs. H batches have by far the highest total pore volume, and the structure changes significantly when any substance is added, reducing the total free volume. The batches with the next highest pore volume contain only SDS, followed by batches containing only cyclodextrins. The batches that combine these two substances have a slightly smaller total volume, which may be explained by the internalization of the SDS in the structure of the cyclodextrins. The batches that contain only polysorbate have hardly any pores, which again agrees with the denser appearance observed previously. The combination of polysorbates and cyclodextrins produces a structure with a greater volume of pores, suggesting that this surfactant, unlike SDS, is unable to internalize in cyclodextrins and cannot therefore occupy the free volume that 2HPβCD leave in the structure of the discs.

Figure 5B,D,F show the pore size distribution. The batches containing HPMC mostly have pores of around 100 μm, consistent with the sizes observed previously when analysing HPMC gels [7]. The addition of any substance other than drugs significantly changes this distribution. A very curious behaviour can be seen in batches containing cyclodextrins or a combination of SDS with 2HPβCD; although the most predominant pores in the structure of these discs originally have a size of nearly 100 μm (Figure 5B), the addition of TFV (Figure 5D) or DPV (Figure 5F) modifies the gel structure to produce discs with pores of about 50 μm. This confirms the ability of cyclodextrins to modify the structural properties of gels and control the porosity of the materials in which they are included and also to form inclusion complexes with the drugs when they are in an aqueous solution [45]. Finally, it should be noted again that the resulting structure has very low porosity when polysorbate is added.

#### 3.2.3. Scanning Electron Microscopy (SEM) Micrographs

**Figure 6:**
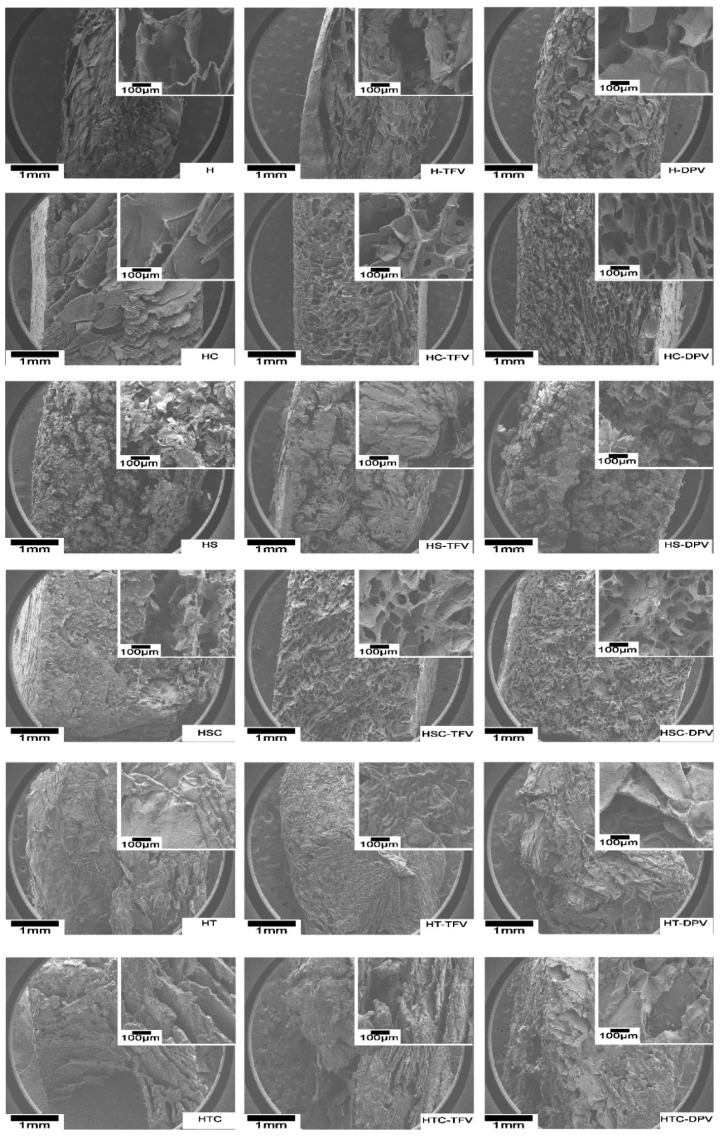
Scanning electron micrographs of vaginal discs.

The inner structure of the discs was observed through SEM (Figure 6). As can be seen in these micrographs, H batches with or without drugs become very structured freeze-dried gels with large pores (around 100μm), which matches the medium pore size observed in Hg porosimetry and is also similar to the structure of other HPMC-based systems [28,46]. The incorporation of 2HPβCD maintains the porous structure although with smaller pores. This phenomenon, i.e., the decrease in pore size with the inclusion of cyclodextrins in the polymer structure, is also observed in the combination with other polymers such as chitosan [47]. When HC batches are loaded with TFV (a water-soluble drug) the pores appear somewhat smaller, and the inclusion of DPV (a poorly water-soluble drug) makes the pores even smaller. These changes in pore size when drugs are included were also noted in porosimetry studies. Batches with only SDS form completely unstructured freeze-dried gels with a fragile appearance. This was also seen in the photographs in Figure 1, but this fragility required confirmation through the analysis of mechanical properties described below. In contrast, batches containing the other surfactant (polysorbate) have a compact appearance with no visible pores (also agreeing with the results for Hg porosimetry and the greater density of these systems).

The analysis of the structure of discs combining cyclodextrins and surfactants reveals some interesting aspects of the formulations. In the combination of 2HPβCD and SDS, it is notable that although no defined structure is observed in the absence of drug, a structure with micropores is formed when TFV or DPV is incorporated. The presence of the drug therefore helps stabilize the interaction between SDS and 2HPβCD. The discs obtained when mixing polysorbate and 2HPβCD in HPMC gels have a completely heterogeneous structure, again revealing the incompatibility of the components.

#### 3.2.4. Mechanical Properties: Deformation and Resistance to Fracture

**Figure 7:**
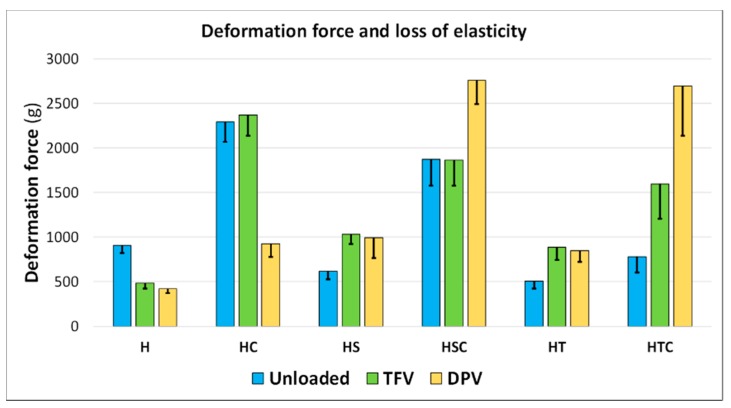
Mean values of the deformation force required to deform vaginal discs a distance of 1 mm (*n* = 4). Inner bars represent the loss of elasticity after 10 deformation cycles.

The mechanical properties of the discs were also evaluated to determine whether they are capable of withstanding the stress involved during their administration. An analysis of the deformability of the discs reveals that the inclusion of a surfactant, either SDS or polysorbate, decreases the deformability of the formulations (Figure 7), whereas the addition of cyclodextrins substantially modifies the structure of the discs, making them much more robust and resistant to deformation, since more than double the force is required to deform them than if cyclodextrins are not included. The combination of 2HPβCD and SDS offers high resistance, although slightly lower than in batches containing only cyclodextrins, while the combination with polysorbate gives the discs a similar resistance to batches that include only surfactants, once again suggesting that 2HPβCD is not properly incorporated into the disc structure. In general, the results confirm the premise that the smaller the size of the pores that form the structure of freeze-dried systems, the greater their deformability [17].

When analysing the influence of drugs, the findings are more disparate. In the reference batches (with only HPMC), the addition of drugs makes the structure more deformable, possibly because the active principle is not uniformly incorporated. In batches with 2HPβCD, this same phenomenon is observed only with the addition of DPV, so TFV can be homogeneously incorporated into the structure when cyclodextrins are present. The addition of surfactants appears to facilitate the incorporation of the drugs, since the complex surfactant-drug forms discs with a more robust structure. The same occurs when surfactants are combined with 2HPβCD, although in these cases, batches that include DPV are particularly notable (HSC-DPV and HTC-DPV) for withstanding the greatest deformation force.

The loss of deformability of the discs is minimal in the batches incorporating only HPMC and increases with the addition of cyclodextrins or surfactants separately; it is even greater in the combination with both solubilizing agents. This is consistent with other studies that evaluate the deformability of freeze-dried systems based on cellulose derivatives, since adding more substances to the systems increases their loss of elasticity [48].

**Figure 8:**
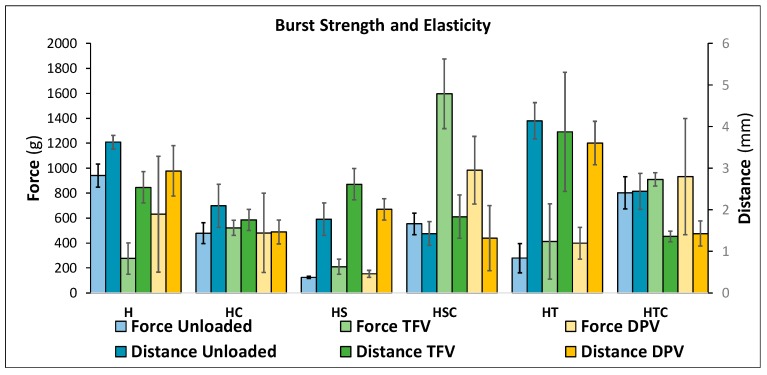
Burst strength and distance to burst of vaginal discs. Mean ± SD are presented (*n* = 4).

The fracture resistance of solid vaginal formulations, whether tablets or films, is always evaluated to ensure that they are suitable for vaginal administration [49,50,51,52]. An analysis was also conducted on the force required to rupture the discs and the distance they are deformed before breaking; the results are shown in Figure 8. It should first be clarified that less force is required to break the discs than to deform them in the previous test, as the probes used to evaluate these parameters are different: a large probe for flat surfaces is used to compress the disc in the deformation test, while the rupture test consists of a puncture made with a probe with a circular head with a small diameter. Since the force is applied to a smaller surface, logically less force is required.

The most notable finding in the fracture resistance test is that the presence of surfactants significantly modifies the mechanical properties of the discs, but in a quite different way. While the addition of SDS makes the systems more fragile so they withstand the least force before breaking (confirming our hypothesis after observing the SEM micrographs that these discs had an unstructured appearance that could cause the fragility of the formulation), the discs become much more elastic when polysorbate is incorporated and are able to deform a greater distance with the application of minimal force. This can also be explained by the results of the previous tests, since the denser and thinner structure of these discs confers better mechanical properties. Other authors report that freeze-dried gels become harder the larger the pore diameter [53], and as can be seen in Figure 8, this is also true in our case, since the reference disc (H batch) withstands the most force before breaking and also has the highest pore diameter.

Another point worth mentioning based on the results is that there are no significant differences between the systems when drugs are incorporated, whatever their nature. However, there is one exception to this statement, and these are the batches that combine 2HPβCD and SDS. As seen in the porosimetry studies (and confirmed in the SEM micrographs), the addition of drugs causes these systems to form a more robust structure with smaller pores. This characteristic also appears to affect the mechanical properties of the discs, since they are capable of deforming a significantly greater distance before breaking, which makes these formulations very suitable for vaginal administration.

#### 3.2.5. Swelling Behaviour

**Figure 9:**
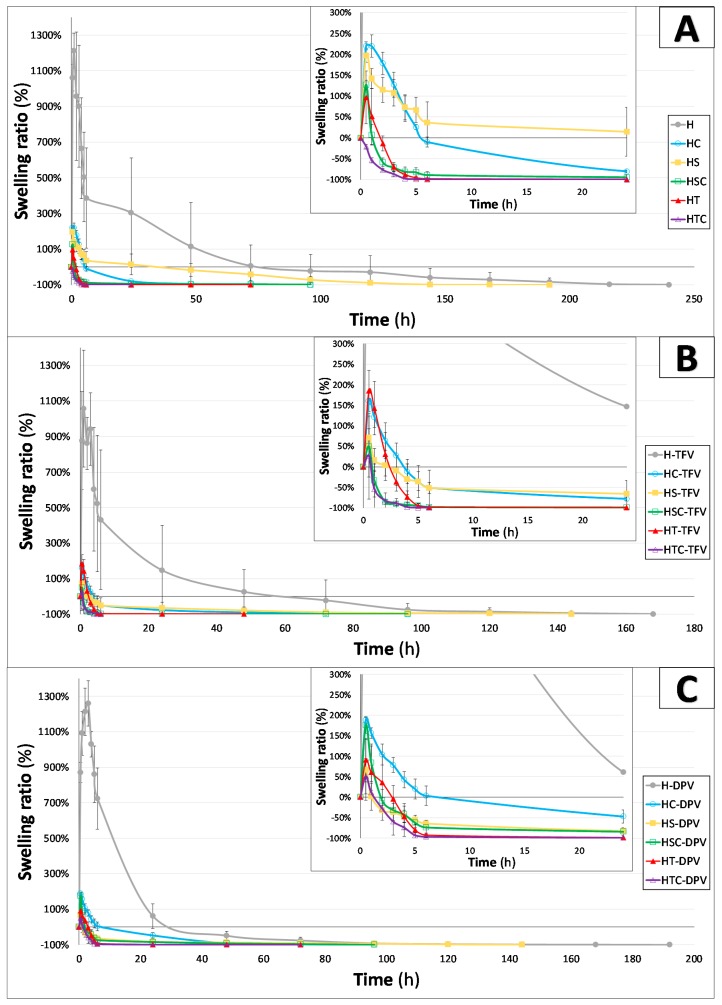
Swelling ratio profile of unloaded (**A**), TFV-loaded (**B**), and DPV-loaded (**C**) vaginal discs immersed in simulated vaginal fluid. The values obtained up to 24 h of trial are expanded inside each picture. Mean ± SD are presented (*n* = 3).

A study was done to determine the behaviour of the discs in the presence of SVF once placed inside the vagina. As this is a freeze-dried system, the discs can be assumed to rapidly capture water after immersion to reconstitute the original gel. As can be seen in Figure 9, this occurs in all batches except those combining polysorbate and cyclodextrins, confirming the incompatibility between these components at the ratios evaluated; they do not form a homogeneous gel, and the freeze-dried system is subsequently unable to rehydrate so the gel can recover at a faster rate than the erosion of the disc. All the other batches swell quickly and reach their maximum swelling ratio at 30 min, except for batches containing only HPMC which have a much more marked swelling and can capture about six times more water than the others. Their maximum swelling ratio is reached 1 h after immersion, which agrees with the results of other authors when evaluating freeze-dried systems based on HPMC [54]. This greater swelling ability is clearly related to the results of the previous test for these batches; since these discs have the highest total pore volume and the largest average pore size, they can absorb a greater amount of SVF. These batches also take longer to completely dissolve, as can be seen in Figure 9.

Similarly, the moderate swelling of the other batches (HC, HS, HSC, and HT) is directly related to the decrease in total pore volume as seen in the porosimetry studies. There are no significant differences between these batches in terms of swelling profiles and almost all are largely dissolved after 24 h. The decrease in the swelling ratio of hydrogels in the presence of cyclodextrins was also expected, not only because of the inability of the 2HPβCD to swell, but also due to the modification of the gel structure [55]. Finally, there are again no significant differences in terms of swelling profile with the addition of drugs to the discs. Only the batches containing only SDS show any difference; when a drug is included in the system, it captures a lesser amount of SVF and is eroded faster. This can be explained by that fact that the complex formed by the drug and the surfactant behaves differently in the presence of the medium than when the SDS is mixed directly in the structure of the HPMC gel.

#### 3.2.6. Drug Release Studies

Undoubtedly the most important feature to evaluate in drug-loaded vaginal discs is the drug release behaviour when the drugs are administered. This was simulated with an in vitro trial, where the discs were immersed in SVF under sink conditions.

**Figure 10:**
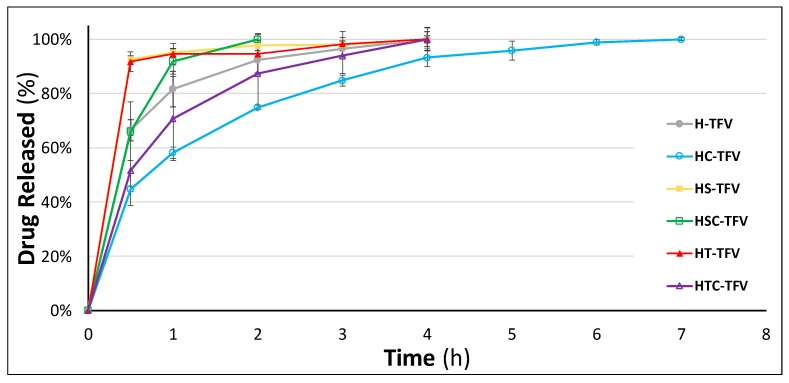
Drug release profiles of TFV-loaded vaginal discs in simulated vaginal fluid. Mean ± SD are presented (*n* = 3).

When analysing the results of the drug release studies for TFV-loaded vaginal discs, the most interesting finding is the control over drug release achieved when 2HPβCD is incorporated in the HPMC structure (Figure 10). These batches are able to release the drug in a sustained manner for 7 h, a notable improvement on the 4 h release time of the reference batch (H-TFV). This proves that the inclusion of cyclodextrins not only contributes to increasing the solubility of drugs but also controls the release of the active principle [56]. In this batch, the control of TFV release is due to the lower swelling ability of the formulation, as seen previously in the swelling test. The smaller pores in its structure hinder the entrance of the medium in the system, thus delaying the TFV diffusion.

In contrast, the addition of a surfactant (SDS or polysorbate) markedly accelerates the dissolution of the drug, and both HS-TFV and HT-TFV batches release around 90% of the drug in 30 min. There are no significant differences in drug release as a function of the surfactant included in the formulation (Table 3). This occurs for two reasons: the higher solubility of TFV in SVF in the presence of the micelles of both surfactants, and the faster erosion of the discs when these substances are included. Nevertheless, in the f2 comparison, it is possible to observe variations among the batches combining these surfactants with the cyclodextrin. This is also appreciable in the drug release graphic; while the combination of SDS with 2HPβCD is capable of achieving the fastest complete release of the drug (in 2 h), the cyclodextrin and polysorbate combination has a similar release profile to the reference batch. However, the HTC-TFV batch shows the highest standard deviation values, undoubtedly due to the erratic release caused by the heterogenicity of this system.

Since DPV is very poorly soluble in SVF, making it difficult to achieve sink conditions, alternative media must be used to evaluate the drug release profiles of DPV-loaded vaginal formulations [57]. Most authors use organic solvents to evaluate the release of DPV [58], but while this may be a good strategy for hydrophobic formulations (such as vaginal rings), it can cause problems when evaluating swellable systems due to the different swelling behaviour in this medium compared to vaginal fluid. However, a good alternative is simulated vaginal fluid modified with the addition of surfactants to increase DPV solubility and achieve sink conditions [59,60]. We have therefore used the medium previously reported by Cazorla-Luna et al., which includes 5% SDS in SVF, to evaluate the DPV release profile from the discs under sink conditions [10].

**Figure 11:**
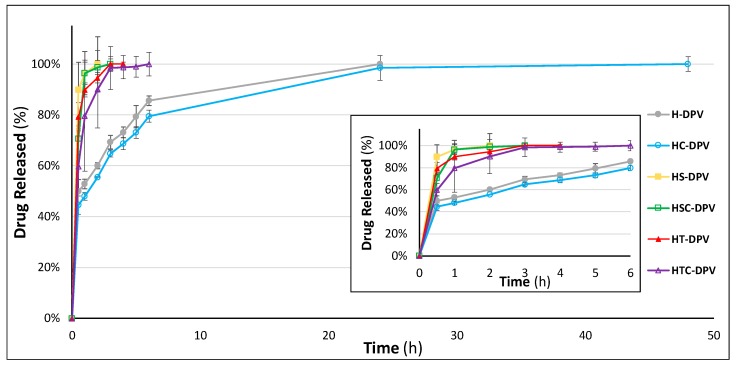
Drug release profiles of DPV-loaded vaginal discs in modified simulated vaginal fluid (including 5% SDS). The values obtained up to 6 h of trial are expanded inside the picture. Mean ± SD are presented (*n* = 3).

As can be seen in Figure 11, batches containing SDS, alone or in combination with 2HPβCD, show the fastest DPV release, with all the drug released in just 2–3 h. Batches including polysorbate (and again their combination with cyclodextrin) also have a very fast release, with all the DPV released in 4–6 h. The reference batch and the disc containing only 2HPβCD (H-DPV and HC-DPV) are therefore capable of moderate drug release, which is sustained for 24 h in both cases. In addition, it is appreciable that there is no statistical difference in the release profile when only the cyclodextrin is included (Table 3). This also confirms that the inclusion of a surfactant notably modifies the structure of the discs as well as improves DPV release, since the micelles of the surfactant favour the dissolution of poorly water-soluble drugs. As can be seen in the statistical analysis, batches HS and HT are similar, although again there are differences when the surfactants are combined with 2HPβCD (HSC and HTC batches). This can be explained due to the different ability to interact with the cyclodextrin of each surfactant agent.

However, as the main aim of including DPV in vaginal discs was to achieve a formulation capable of helping DPV to fast-release in the presence of vaginal fluid, the batches were also evaluated in SVF to observe how they would release the drug in the vaginal environment.

**Figure 12:**
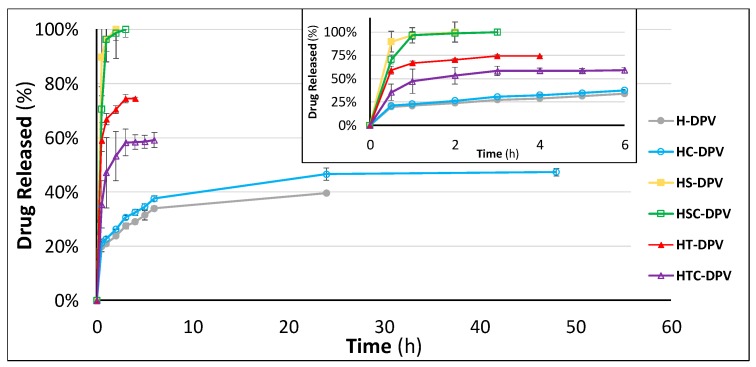
Drug release profiles of DPV-loaded vaginal discs in simulated vaginal fluid. The values obtained up to 6 h of trial are expanded inside the picture. Mean ± SD are presented (*n* = 3).

This drug release study reveals no significant differences when cyclodextrin is included (HC-DPV) compared to the reference batch (H-DPV) (Table 3). This confirms that DPV is unable to form inclusion complexes with 2HPβCD, so the solubility is not improved: none of these batches are able to release more than 2.5 mg of DPV after 48 h (Figure 12).

Nevertheless, each of the other batches is significantly different, as is observed in the f_2_ comparison. The addition of polysorbate increases the total amount of drug released, but is still unable to completely dissolve the 5 mg of drug in the formulation. It is striking that although the total amount of surfactant is the same for HT-DPV and HTC-DPV batches, the final drug dissolved is lower when cyclodextrins are included. The aforementioned incompatibility between P60 and 2HPβCD causes the polysorbate micelles not to exhibit their solubilising power, which is why the drug release profile from HT-DPV is greater than that from HTC-DPV.

In contrast, the inclusion of SDS achieves the complete dissolution of the DPV in the disc, so it is potentially a good strategy to achieve a DPV-loaded formulation capable of fast release once administered vaginally. The combination of SDS with 2HPβCD shows the same drug release profile, but it is worth highlighting the usefulness of including cyclodextrins; due to the vaginal cytotoxicity of free SDS (it has already be proven that high concentrations of this surfactant can cause vaginal ulcers), it is imperative to evaluate the toxicity of any formulation that includes this substance before testing in clinical trials [61]. Since it has been demonstrated that SDS is able to form a complex with 2HPβCD, this complex is also probably less cytotoxic than when SDS is in a free state, as occurs in the combination of cyclodextrins with other cytotoxic substances [62], so the inclusion of cyclodextrins in this system may be valuable, not in terms of drug release, but from a safety point of view. Obviously, the amount of SDS included in the discs must also be adjusted and reduced to the minimum required for drug solubility.

#### 3.2.7. Mucoadhesion Time and Force

Finally, the adhesive properties of the vaginal discs were also evaluated in order to demonstrate that the discs can be retained in the vagina after their administration, since their possible expulsion would lead to the subsequent failure of the formulation to protect against infection.

**Figure 13:**
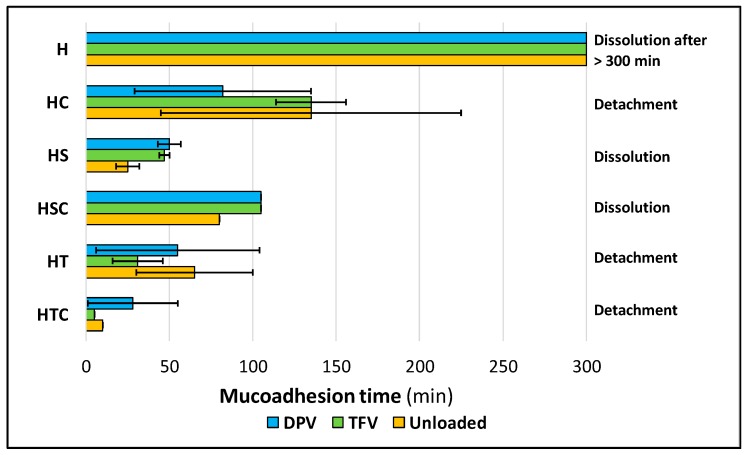
Mucoadhesion time until the detachment of the vaginal discs. At right is detailed if mucoadhesion time is limited by the detachment or complete dissolution of the disc. Mean ± SD are presented (*n* = 2).

Mucoadhesion time was evaluated, and the results are shown in Figure 13. It should first be mentioned that only batches H, HS, and HSC (as well as their drug-loaded equivalent batches) are capable of remaining adhered until their complete dissolution. Batches containing only 2HPβCD, only polysorbate, or a combination of both, detached before they were completely dissolved. The reference batches (containing only HPMC) have by far the longest mucoadhesion times, undoubtedly due to their higher swelling ratio, which is directly related to the mucoadhesive properties [51,63]. 

The fact that the addition of SDS causes the system to remain adhered until its complete dissolution is because it is an anionic surfactant that enables the discs to bond to the mucosa due to electrostatic charges and with hydrogen bonds formed by HPMC [64,65]. In contrast, the inclusion of 2HPβCD scarcely modifies the disc structure so it also retains most of the adhesive potential of HPMC; these batches have the second longest mucoadhesion time after the reference batches. However, possibly the most suitable batches in terms of mucoadhesion time combine SDS and cyclodextrins, since they still have a good mucoadhesion time (due to the presence of 2HPβCD) and can also remain attached to the vaginal mucosa until complete dissolution (due to the presence of SDS). It should also be noted that there are no significant differences in mucoadhesion time when the drugs are included in the formulations.

**Figure 14:**
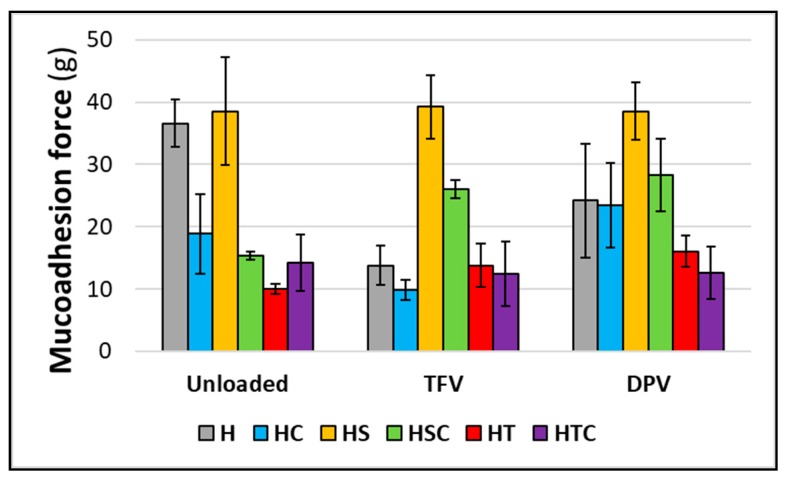
Mucoadhesion force required to detach the vaginal discs. Mean ± SD are presented (*n* = 3).

However, when evaluating mucoadhesive force, greater adhesiveness is observed in batches containing only SDS (Figure 14). This is because of the bonding by electrostatic charges, which are stronger than hydrogen bonds [7]. This is statistically demonstrated by means of paired t-test analysis, where it is observed that HS batches differ from batches with cyclodextrin, polysorbate, and their combination (Table 4). Again, the combination of SDS and 2HPβCD shows an adequate mucoadhesion force, which is even greater than the force obtained in reference batches when TFV or DPV is included in the vaginal discs. No significant differences are observed when unloaded batches are compared with batches containing any drug, so it can be concluded that the addition of drugs to the discs barely modifies their mucoadhesive properties (Table 4).

Therefore, while HS batches are notable for their excellent mucoadhesion force, HC batches stand out for their adhered residence time. Thus, HSC batches are the most adequate in terms of mucoadhesion, as they have an acceptable adhesion force and are able to remain adhered until their complete erosion.

In view of the results, it can be confirmed that vaginal discs have great potential for vaginal administration of microbicides. Although the current trend is to search for dosage forms that allow a sustained release of drugs in order to stagger the administration interval, another alternative for better patient compliance is to develop coitally-dependent formulations that are administered by women just before intercourse and offer immediate protection.

TFV-loaded discs clearly achieve this objective, as with the inclusion of surfactants they are able to release practically all the drug in only half an hour. The formulation also proved to be useful for the rapid release of a lipophilic drug, DPV, despite its poor solubility in the vaginal environment.

Another advantage of vaginal discs compared to other vaginal dosage forms comes from their unlikely interference with intercourse. It is clear that solid dosage forms—especially vaginal rings— can be detected by men during intercourse and can cause discomfort [66]. Moreover, vaginal gels usually cause leakage and messiness. Nevertheless, vaginal discs would capture the volume of fluid already present in the vagina to form a gel, so the formulation would not result in an additional volume in the vaginal environment. This behaviour is similar to that already observed in vaginal films [67]. In addition, the volume the discs capture—as observed in the swelling test—would be lower than 2 mL, so the volume can be considered acceptable. Finally, it should also be mentioned that the stress during intercourse will not only affect the efficacy of the formulation but could even accelerate the dissolution of the system. The inclusion of cyclodextrins in these formulations performs numerous functions: 2HPβCD modulates the release of TFV, significantly improves the mechanical properties of the formulations (without which they would be too fragile to administer), and particularly increases the adhesiveness of the formulation to the vaginal mucosa, which is essential for it to be retained in the vaginal environment until all of the drug is released. If we compare the adhesiveness force of the developed formulations to vaginal films developed for the administration of antiretroviral drugs, we can appreciate that the force required to detach both formulations from the vaginal mucosa is really similar [68]. Cyclodextrins are also considered to play a fundamental role in counteracting the cytotoxicity of SDS.

Future studies are needed to adjust the dose of drug administered, as the discs have been shown to hold and release large drug doses, 10 mg of TFV and 5 mg of DPV, and consequently, the amount of surfactant and cyclodextrins necessary to reduce the possible toxicity to safe levels.

## 4. Conclusions

Vaginal discs based on HPMC freeze-dried gels are a pharmaceutical form suitable for the fast release of vaginally administered drugs.

The inclusion of SDS in HPMC gels allows DPV (a poorly water-soluble drug) to be incorporated and dissolved in the vaginal medium at the same rate as TFV (a hydrophilic drug).

The combination of SDS and 2HPβCD modifies the microstructure of the HPMC gel and produces freeze-dried systems with smaller pores, forming a final structure with a lower pore volume, which not only allows the inclusion and release of over 90% of the drugs, both TFV and DPV, in just half an hour, but also creates a robust structure that gives the discs greater mechanical strength and better mucoadhesive properties. This fast release would guarantee rapid protection after placing it in the vagina.

The administration of vaginal discs based on HPMC freeze-dried gels immediately prior to sexual intercourse could offer women a comfortable tool for protection against the sexual acquisition of HIV. The comfortable administration, and especially the on-demand use, may be translated into better adherence to the use of this microbicide by women, which has been the main problem observed to date with vaginal microbicides.

## Figures and Tables

**Table 1:** Composition of vaginal discs (mg).

Group	Batch	Hydroxypropylmethyl Cellulose	2-Hydroxypropyl-β-Cyclodextrin	Sodium Dodecyl Sulphate	Polysorbate 60	Tenofovir	Dapivirine
Unloaded	H	80					
	HC	80	400				
	HS	80		400			
	HSC	80	400	400			
	HT	80			400		
	HTC	80	400		400		
TFV-loaded	H-TFV	80				10	
	HC-TFV	80	400			10	
	HS-TFV	80		400		10	
	HSC-TFV	80	400	400		10	
	HT-TFV	80			400	10	
	HTC-TFV	80	400		400	10	
DPV-loaded	H-DPV	80					5
	HC-DPV	80	400				5
	HS-DPV	80		400			5
	HSC-DPV	80	400	400			5
	HT-DPV	80			400		5
	HTC-DPV	80	400		400		5

**Table 2:** *p*-Values obtained in the comparison of the group of batches by a paired *t*-test. Significant differences are in bold.

Reference Group	Problem Group	*p* Value (α = 0.05)	
		Penetration Work	Detachment Work
Unloaded	TFV-loaded	0.2083	0.4193
Unloaded	DPV-loaded	0.1798	0.8829
TFV-loaded	DPV-loaded	0.2647	0.3584
H	HC	**0.0151**	0.0547
H	HS	**0.0028**	**0.0142**
H	HSC	**0.0016**	0.2025
H	HT	**0.0134**	**0.0124**
H	HTC	0.0640	**0.0138**
HC	HS	0.3661	**0.0013**
HC	HSC	**0.0112**	**0.0120**
HC	HT	**0.0495**	**0.0015**
HC	HTC	0.1234	**0.0016**
HS	HSC	**0.0176**	**0.0011**
HS	HT	**0.0328**	0.9020
HS	HTC	**0.0235**	0.0654
HSC	HT	0.2105	**0.0007**
HSC	HTC	**0.0197**	**0.0007**
HT	HTC	**0.0107**	**0.0052**

**Table 3:** Similarity factor (f_2_) values obtained from the comparison of batches. Significant differences are in bold.

Reference	Problem	f_2_ Value		
		TFV-loaded SVF	DPV-loaded SVF + SLS	DPV-loaded SVF
H	HC	**37.0**	65.1	72.5
H	HS	**36.7**	**27.5**	**15.3**
H	HSC	61.0	**27.8**	**14.0**
H	HT	**37.4**	**28.2**	**19.3**
H	HTC	51.0	**34.2**	**30.1**
HC	HS	**23.6**	**24.7**	**15.7**
HC	HSC	**31.9**	**24.9**	**14.5**
HC	HT	**24.0**	**25.1**	**20.6**
HC	HTC	51.0	**30.4**	**32.5**
HS	HSC	**36.3**	**43.3**	**43.3**
HS	HT	96.6	56.1	**33.1**
HS	HTC	**26.9**	**33.6**	**20.7**
HSC	HT	**37.0**	60.0	**36.9**
HSC	HTC	**41.5**	**46.9**	**22.8**
HT	HTC	**27.4**	**44.8**	**38.3**

**Table 4:** *p*-Values obtained in the comparison of the group of batches by paired t-test. Significant differences are in bold.

Reference Group	Problem Group	*p*-Value
Unloaded	TFV-loaded	0.5441
Unloaded	DPV-loaded	0.6698
TFV-loaded	DPV-loaded	0.1095
H	HC	0.2851
H	HS	0.1769
H	HSC	0.8837
H	HT	0.2778
H	HTC	0.1916
HC	HS	**0.0364**
HC	HSC	0.4117
HC	HT	0.4179
HC	HTC	0.3869
HS	HSC	0.0577
HS	HT	**0.0047**
HS	HTC	**0.0008**
HSC	HT	**0.0492**
HSC	HTC	0.1563
HT	HTC	0.9514
